# Obituary: Professor Paul Dan Cristea

**DOI:** 10.1186/1687-4153-2013-7

**Published:** 2013-05-10

**Authors:** Ioan Tabus, Erchin Serpedin, Jaakko Astola

**Affiliations:** 1 Department of Signal Processing, Tampere University of Technology, Korkeakoulunkatu 1, Tampere, FIN-3310, Finland; 2 Department of Electrical Engineering, Texas A&M University, College Station, TX 77843-3128, USA

## Abstract

Paul Dan Cristea, professor of Electrical Engineering and Computer Science at ‘Politehnica’ University of Bucharest died on 17 April 2013, following several years of bravely battling a perfidious illness.

## 

Paul Cristea was born in Bucharest on February 13, 1941. Paul graduated with two master degrees: one from Polytechnic Institute of Bucharest, Department of Electronics and Telecommunications, Physics Engineering Division, as head of his 1962 series, and one from the University of Bucharest, Department of Physics. He obtained Ph.D. degree in 1970 from the Polytechnic Institute of Bucharest. After graduation he embraced the university career, advancing through the whole range of university positions from assistant professor in 1962 to full professor in 1990.

He was a very talented researcher, having an extremely wide range of research interests and bringing contributions to many fields such as physics, electrical engineering, and bioinformatics. He founded the Digital Signal Processing Laboratory at the ‘Politehnica’ University of Bucharest, where he pursued during the last two decades timely research on hot subjects such as wavelet image processing, neural networks, and bioinformatics. One of the research topics that spanned throughout his whole career was the study of the properties of DNA sequences, ranging from various mathematical representations of DNA sequences and going to the development of techniques through which to extract meaningful information from the DNA sequences at different scales. He deployed a pioneering activity in the study of biological sequences via signal processing methods, and he was one of the main promoters of the genomic signal processing field.

Paul was a tireless advocate of the need for joint research work and systematic collaboration of the engineers from areas like signal processing and computer science, with mathematicians, biologists, and medical scientists for uncovering the hidden rules and laws that govern the wealth of data and information provided by the life sciences technologies. He worked hard to promote the importance of genomic signal processing as a new interdisciplinary area and to create multi-disciplinary research groups first in Romania and later, with teams from many European universities. Therefore, many signal processing research groups in Europe have been attracted in joining forces for developing the genomic signal processing field. Paul also organized several workshops on genomic signal processing, which became important meeting places for researchers to share their latest results and to extend the research activities in the emerging field of genomic signal processing.

Paul served as an associate editor and member of the board of the *EURASIP Journal of Bioinformatics and System Biology* from the foundation of the journal in 2005. He was a very active member of the Editorial Board, proposing and reviewing many special issues of the journal. Paul also served on the editorial boards of a dozen of journals and as an investigator for about 60 research projects.

Paul's contribution to science and engineering was not confined only to his deep research work. He was a generous educator who helped tens of M.Sc. and Ph.D. students at the ‘Politehnica’ University of Bucharest to graduate. He was a very dedicated teacher whose lectures were very clear and well appreciated by his students. Paul deployed also a very prolific activity as a writer. He wrote about 20 textbooks and research monographs (mostly in Romanian) that were very well received and appreciated by students, engineers, and the whole academic community in Romania. Paul is also the author of about 100 journal papers, 400 conference papers and 11 patents. However, above all these accomplishments, Paul was a creator of communication bridges with tens of prestigious universities in Europe and USA, where he sent many of his students as exchange students, mostly of them graduating at their new host universities and becoming themselves very respected researchers.

Paul was a great researcher and dedicated teacher who passed the burning torch of his passion for science to his students and collaborators simply by starting to talk about his favorite subjects, those for which he dedicated long days and nights of research either in his laboratory or in front of his home computer. He gave invited lectures in Finland, Greece, Belgium, Netherlands, Japan, and USA.

Paul was also a human being with a rich culture and generous spirit. He was also a good friend. He was always leading the discussions with friends and students towards extraordinary and amazing subjects, discussing with passion and deep knowledge on a large number of favorite science subjects, but also with a charming eloquence telling one thousand and one stories about the many places he had visited and explored for the unusual. He had a very good sense of humor both in casual conversations and also when teaching, making all subjects attractive and accessible for both uninitiated and scholars.

His activity was rewarded with many honors, including election as a member of Romanian Academy and Fellow of IEEE. He was an active evaluator of the research projects in EU framework, and he was also a very active coordinator of EU research and networking projects.

His wife and two daughters survive him. He was always very proud that his passion towards science and excellence was passed on to his daughters, who are currently accomplished researchers and educators. He will be sorely missed by generations of students, by his many peers, friends, and collaborators in the scientific community.

## Notes

On behalf of the Editorial Board of *EURASIP Journal of Bioinformatics and System Biology*.

Ioan Tabus

Jaakko Astola

Erchin Serpedin

